# Polarization induced two dimensional confinement of carriers in wedge shaped polar semiconductors

**DOI:** 10.1038/srep26429

**Published:** 2016-05-23

**Authors:** S. Deb, H. P. Bhasker, Varun Thakur, S. M. Shivaprasad, S. Dhar

**Affiliations:** 1 Department of Physics, Indian Institute of Technology Bombay, Powai, Mumbai 400076, India; 2 International Centre for Material Science, Jawaharlal Nehru Centre for Advanced Scientific Research, Bangalore 560064, India

## Abstract

A novel route to achieve two dimensional (2D) carrier confinement in a wedge shaped wall structure made of a polar semiconductor has been demonstrated theoretically. Tapering of the wall along the direction of the spontaneous polarization leads to the development of charges of equal polarity on the two inclined facades of the wall. Polarization induced negative (positive) charges on the facades can push the electrons (holes) inward for a n-type (p-type) material which results in the formation of a 2D electron (hole) gas at the central plane and ionized donors (acceptors) at the outer edges of the wall. The theory shows that this unique mode of 2D carrier confinement can indeed lead to a significant enhancement of carrier mobility. It has been found that the reduced dimensionality is not the only cause for the enhancement of mobility in this case. Ionized impurity scattering, which is one of the major contributer to carrier scattering, is significantly suppressed as the carriers are naturally separated from the ionized centers. A recent experimental finding of very high electron mobility in wedge shaped GaN nanowall networks has been analyzed in the light of this theoretical reckoning.

Two dimensional (2D) carrier gas (2DCG), where the carriers are virtually confined in a 2D sheet, offers a test bed to explore new directions in physics by giving rise to many remarkable phenomena such as several orders of magnitude enhancement of mobility, integer and fractional quantum hall effects^1^,^2^,^3^, 2D metal insulator transition^4^ and microwave induced zero resistance state^5^. The system thus remains to be the subject of intense research for several decades. Most of the techniques to achieve 2D confinement of carriers involve semiconductors. The common approach is to confine carriers either in a triangular potential well formed at the heterointerface between two semiconductors or in a rectangular well formed by sandwiching a lower band gap semiconductor layer between higher band gap semiconductors^6^. These heterostructure based 2D systems made of only a few limited semiconducting materials, mainly GaAs, are extensively investigated so far. Many of the fascinating results mentioned above are in fact observed in these systems. Recently, new classes of 2D systems have opened up novel aspects of 2D carriers. One example is the formation of high electron mobility 2D electron gas (2DEG) at the interface between two insulating oxides – LaAlO_3_ and SrTiO_3_^7^. The 2DEG, in this case, arises as a result of electron entrapment in certain exotic interface states, which are not possible to realize in bulk. Another example is graphene, where 2D carrier gas is formed as a result of atomically thin layer width^8^,^9^. In graphene, electrons behave like massless charge particles due to linear dispersion in the band profile. An entirely different route for 2D confinement of carriers can be thought about in a wedge shaped wall structure made of a polar semiconductor. Gradual narrowing of the wall along the direction of the spontaneous polarization can lead to a unique situation, where the charges of equal polarity develop on the two inclined facades of the wall. For the case of negative(positive) charge accumulation on the side facades, electron(hole) cloud in a n-type(p-type) material is pushed inward, resulting in the formation of a 2D electron(hole) gas at the central plane of the wall. This furthermore leads to the formation of positive(negative) depletion regions on the both sides of the 2D confinement. This natural way of partitioning of the electrons(holes) from the ionized donors(acceptors) is expected to reduce significantly the ionized impurity scattering, which is one of the major factors for the reduction of mobility. The question remains whether such a scenario is at all feasible or not.

Here, we have chosen GaN, a strongly polar semiconductor as a test case. Schrödinger and Poisson equations are solved self-consistently to obtain the potential and charge density distribution within a n-type GaN nanowall that is tapered along its polarization direction. The result indeed supports the formation of a 2D electron gas in the central vertical plane of the wall. Moreover, the low field electron mobility in 2DEG has been theoretically calculated taking into account three major scattering contributions namely the ionized-impurity, neutral-impurity and polar-optical phonons. The study reveals several orders of magnitude enhancement of electron mobility in this system at all temperatures as compared to bulk. Finally, a recent experimental finding of very high electron mobility in wedge shaped GaN nanowall networks has been examined in the light of these theoretical results.

## Formation of 2D electron gas in wedge shaped GaN wall structure

**Figure 1:**
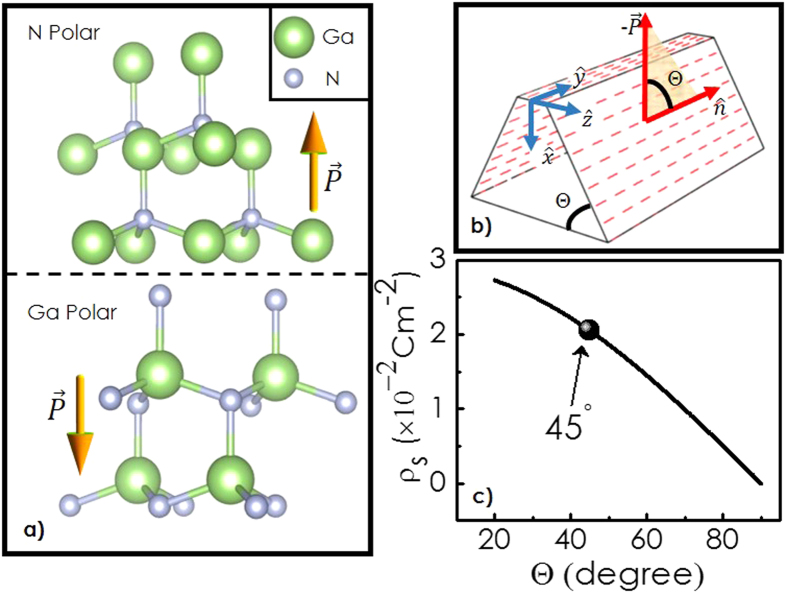
(**a**) Arrangement of Ga-N bonds and the direction of polarization in case of N-polar and Ga-polar GaN epitaxial layer. Here VESTA 3 is used for 3D visualization. (**b**) Schematic representation of a c-oriented wedge shaped Ga-polar GaN wall structure. Polarization along c-direction induces negative surface charges (represented by red dashes) on the flat top surface as well as on the two facades inclined at an angle Θ with respect to the base. (**c**) Polarization induced negative surface charge density at the two inclined facades as a function of Θ.

GaN has a wurtzite crystalline structure, in which each Ga(or N) ion is connected with four nitrogen(or Ga) ions in tetrahedral coordination. An epitaxial GaN film, when grows on a substrate along its c-direction, can have one of the two distinctly different orientations of Ga-N bonds. The layer, in which Ga-N single bond along the c-axis runs with N on top of Ga, is termed as Ga-polar GaN. While in N-polar GaN films, Ga-N single bond along the c-axis runs with Ga on top of N. This is shown in Fig. 1(a). GaN has a spontaneous polarization 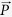 (=0.029 Cm^−2^)^10^ along c-direction. For a Ga-polar GaN layer 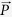 runs downward, making the top and bottom surfaces of the film negative and positive, respectively. An interesting situation arises if instead of a flat film, a wedge shaped wall structure is grown along c-axis, which has a gradual tapering along the growth direction as shown in Fig. 1(b). Note that the polarization induced surface charge density on a facade can be expressed as 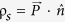, where 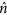 is the unit vector normal to the surface. In case of a Ga-polar GaN wall, both the inclined facades acquire negative surface charge density. Figure 1(c) shows the variation of ρ_*s*_ as a function of the inclination angle Θ. For n-type GaN, negative charges on the side facades are likely to push the electron cloud from two sides forcing them to confine in the central vertical plane of the wall.

In order to calculate the potential and charge density distribution inside such a wall one has to solve Poisson’s and Schrödinger equations self consistently. Here, we have considered that the potential is invariant along y-direction. The Poisson’s and Schrödinger equations are thus solved self-consistently in a 2D (in xz-plane) space confined within a trapezoidal boundary (cross-section of a wedge shaped nanowall). The Poisson’s equation is written as:


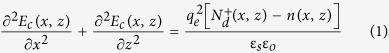


where, *z* represents the coordinate along the wall width [see Fig. 1(b)]. *E*_*c*_(*x, z*) is the position of the conduction band minimum and *q*_*e*_ the electronic charge. 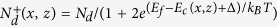 is the ionized donor distribution at temperature *T* with Δ the donor activation energy, *N*_*d*_ the donor concentration, *k*_*B*_ the Boltzmann constant and ε_*s*_ the low frequency dielectric constant of GaN. *E*_*f*_ the chemical potential, commonly termed as Fermi energy. 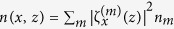 the spatial distribution of electron density, where 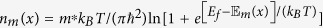 is the carrier density in the m-th subband as a function of x, m* the electron effective mass and *ħ* the reduced Planck’s constant. Here, 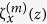 is the z-dependent part of the wavefunction associated with the eigenvalue 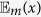. It is noteworthy that the wavefunction, in this case, can be expressed as 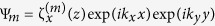 considering the electron to be free along x and y-directions. Furthermore, since *E*_*c*_ is expected to be a slowly varying function of *x*, the Schrödinger equation can be approximated as a set of 1D (z-dependent) Schrödinger equations each of which is associated with a specific *x* position as given below^11^


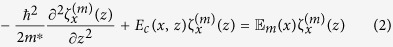


Note that the involvement of electronic wave function on the source term of Poisson’s equation makes it necessary to solve these two equations self consistently. Equations 1 and 2 are solved numerically^12^,^13^ in a self-consistent manner keeping in mind that the solution must satisfy the following boundary conditions: 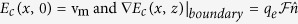. Where, 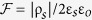 is the electric field at the boundaries resulting from the polarization charges. 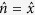 for the top flat surface and 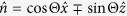 for the right and left inclined surfaces of the wall. Here, the polarization charge density ρ_*s*_ at the bottom surface has been neglected [polarization charges at the bottom surface is often canceled/suppressed by the charges of opposite polarity resulting from the substrate polarization]. Here, the inclination angle Θ of the wall has been taken to be 45°, which corresponds to a surface charge density at the inclined planes of the wall to be ρ_*s*_ = −0.0205 Cm^−2^. Width of the base and the top flat surface of the wall are considered to be 100 and 10 nm, respectively. Donor concentration *N*_*d*_ and activation energy Δ has been taken to be of 1 × 10^19^ cm^−3^ and 15 meV, respectively^14^. More details about the numerical procedure adopted here for these calculations has been provided in supplementary S-1.

**Figure 2:**
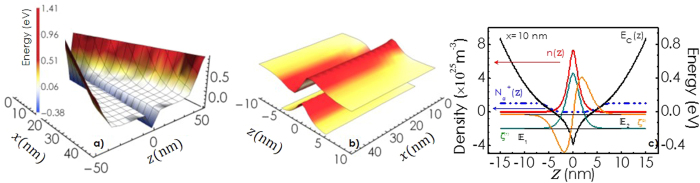
(**a**) A 3D-plot for *E*_*c*_ in *xz*-plane obtained by solving Schrödinger and Poisson’s equations self consistently as discussed in the text. (**b**) 3D-plot for the self-consistent ground and the first excited eigenstates 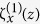 and 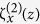 (**c**) Cross-sectional view of the self-consistent solutions at *x* = 10 nm for the *E*_*c*_ profile, energy eigen states, eigenvalues, as well as electron concentration and ionized donor density profiles.

**Figure 3:**
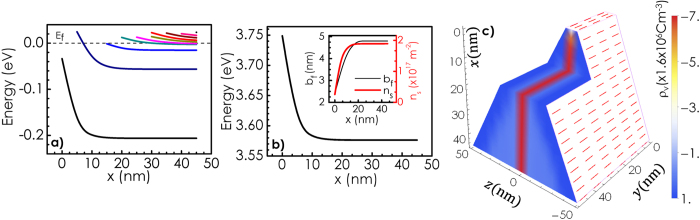
(**a**) Variation of energy eigen values with *x*. (**b**) Band gap energy 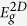 at the 2DEG region as a function of *x*. Inset shows the dependence of confinement width *b*_*f*_ and electron density *n*_*s*_ of the 2DEG on *x*. (**c**) 3D color plot for the charge density ρ_*v*_(*x, y, z*) distribution inside the wedge shaped c-oriented GaN wall. Negative and positive regions are represented by red and blue colors respectively. Evidently, electrons are confined in a thin sheet located at the central vertical plane of the wall. A portion of the wall has been artificial cut open in order to give a better feeling about the extent of the electron rich central plane.

A 3D-plot for *E*_*c*_ in *xz*-plane is shown in Fig. 2(a). In these calculations, Fermi level is considered to be at zero energy. Evidently, a triangular potential well is formed at the central plane, where *E*_*c*_(*z*) goes below the Fermi level. Most striking outcome of our calculation however is the quantum confinement of the electrons at the central part of the potential profile. 3D-plots for the self-consistent solutions for the ground 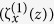 and the first excited state 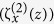 are shown as a function of *x* and *z* in Fig. 2(b). Figure 2(c) showcases the electron concentration and ionized donor density as a function of *z* for *x* = 10 nm. Note that the electrons are tightly concentrated at the middle of the potential profile, while the ionized donors are distributed at the boundaries. Figure 3(a) illustrates the variation of the energy eigenvalues 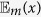 as a function of *x*. Though, the total number of eigenstates (calculated up to *k*_*B*_*T* energy above *E*_*f*_) increases with *x*, number of states lying below the Fermi level is not crossing 4 even at the base. Interestingly, the separation between energy eigen states and the Fermi level increases initially with *x* but saturates later. This can be attributed to the increase of sheet carrier density in 2DEG *n*_*s*_ with *x*, which has been shown in the inset of Fig. 3(b). Figure 3(b) shows the variation of the band gap energy 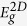, which is the separation between the lowest energy eigen state and the valence band maximum for the 2DEG region, as a function of *x*. Note that 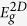 is more than 300 meV blue shifted at the tip of the wall as compared to the band gap energy of 3.4 eV for GaN bulk (see supplementary information S-1). The width of the electron confinement *b*_*f*_(*x*) defined as the width at which the charge density becomes one fifth of its maximum value has also been plotted as a function of *x* in the inset of Fig. 3(b). Note that both *b*_*f*_(*x*) and *n*_*s*_(*x*) show a rapid enhancement followed by a saturation as *x* increases. Figure 3(c) shows the 3D color plot for the charge density ρ_v_(*x, y, z*) distribution inside the wedge shaped c-oriented GaN wall. ρ_v_ is considered to be invariant along *y*-axis. Evidently, electrons are confined within a thin sheet (red coloured region) located in the central plane of the wall. It is worth noting that as the width of the wall increases (as *x* increases), the region where the band profile is flat [light blue region] grows much faster than the region where the quantum confinement takes place. Bulk like property is thus expected to overwhelm the characteristics of the quantum confinement at the bottom part of the wall.

These results clearly demonstrate that a c-axis oriented wedge shaped GaN wall can indeed serve as a system where spontaneous polarization leads to 2D confinement of electrons, an entirely new route to get 2DEG in a semiconductor structure. This method has certain advantages over the conventional heterostructure based techniques: Firstly, growth of two different materials one on top of another (heteroepitaxy) is not required in this technique. Secondly, the electron cloud, which is tightly confined at the center is spatially separated from the ionized donors concentrated at the boundaries. This natural way of separation is expected to reduce the ionized impurity scattering, which is one of the important factors for the suppression of mobility. This has encouraged us to theoretically estimate the electron mobility in the 2DEG channel formed in this way.

## Calculation of mobility

Mobility associated with a given scattering process *i* can be expressed as^15^


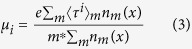


where, the average momentum relaxation time for the electrons belonging to the *m*-th subband, *τ*^*i*^(*E*_*m*_) the momentum relaxation time for a given energy *E*_*m*_ and 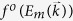 the Fermi distribution function (see supplementary S-2 for detail). Note that the total energy associated with *m*-th subband , where and 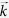 the wave-vector in *xy*-plane. Finally the total mobility is calculated using Matthissen’s rule; 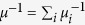. We have taken into account the ionized impurity (II)^16^,^17^,^18^,^19^,^20^, neutral impurity (NI)^21^,^22^ and polar optical phonon (PO)^23^,^24^,^25^ scattering processes as dominant mechanisms in governing the total mobility of the system. It should be noted that inter-subband scattering processes are neglected for ionized impurity and neutral impurity scattering cases, while they are taken into account for scattering by polar optical phonons. Note that in case of elastic scattering processes (ionized and neutral impurity scattering) the inter-subband scattering can be neglected for the lower sabbands as their energy separations [see Fig. 3(a)] are too large for such scattering to take place. Though, the same is not true for higher lying subbands, which become relevant for the bottom part of the wall. Since these subbands are energetically close to each other, inter-subband scattering processes are not negligible but the electron population in these states as compared to that of the ground state is expected to be too less to influence the overall mobility significantly. However, in case of polar optical phonon scattering, inter-subband scattering can not be neglected as the energy of these phonons (≈90 meV) is high enough to support inter-subband transition even for the lower sabbands. It is also worth mentioning that involvement of 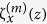 in *τ*^*i*^(*E*_*m*_) will eventually impose an additional *x* dependency on *μ*_*i*_. A detailed discussion on the calculation of mobility is provided in the supplementary S-2.

**Figure 4:**
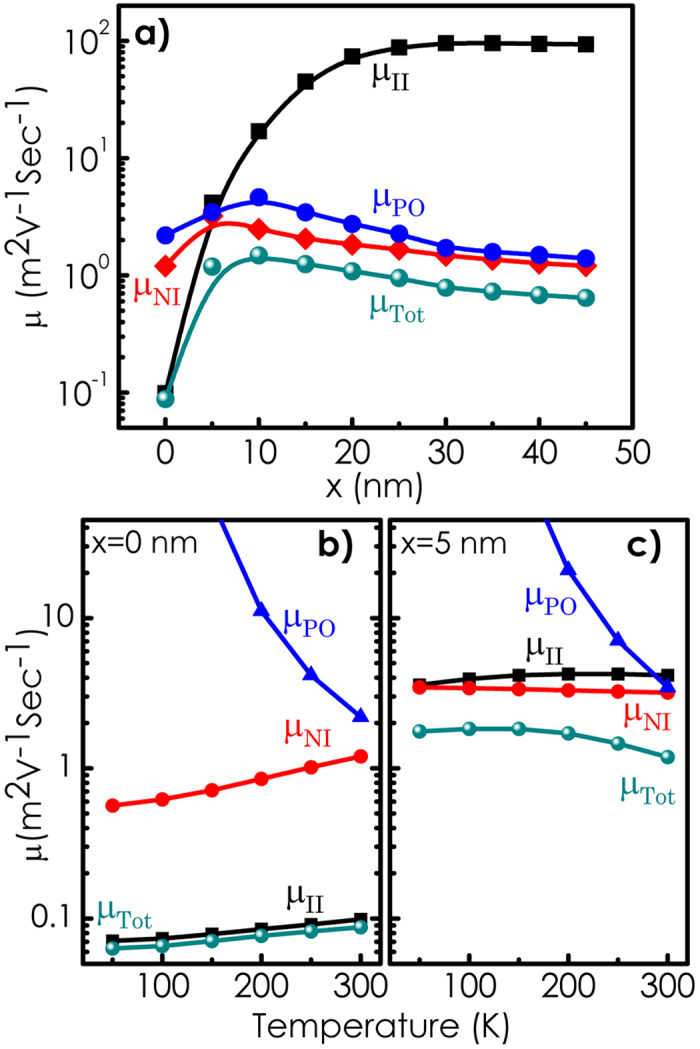
Total electron mobility *μ* along with the contributions associated with individual scattering processes calculated (**a**) at 300 K as a function of *x* and as a function of temperature for (**b**) *x* = 0 nm and (**c**) *x* = 5 nm. Overall mobility shows a weak temperature dependence.

Here, ionized and neutral donors are considered to be the only centers for ionized and neutral impurity scattering, respectively. Figure 4(a) shows the dependence of electron mobility *μ* calculated at 300 K as a function of *x*. Contributions associated with individual scattering processes are also shown as a function of *x* in the figure. Most interestingly, the calculated mobility comes out to be several orders of magnitude larger than what is expected for GaN bulk^26^ with such a high donor concentration especially for lower values of *x*. Notably, the mechanism for scattering here is dominated neither by ionized impurities nor by polar optical phonons. Neutral impurities play the most important role. As *x* increases, mobilities associated with neutral impurity (*μ*_*NI*_) and polar optical phonon scattering (*μ*_*PO*_) show an increase followed by a gradual reduction. On the other hand, mobility associated with ionized impurity scattering *μ*_*II*_ monotonically increases with *x* and becomes almost independent of *x*. Initial increase of mobility with *x* for all three cases can be attributed to the enhancement of Fermi energy with respect to the ground state as *x* increases [see Fig. 3(a)]. Note that in case of ionized impurity scattering, the increase of mobility with *x* can partly be attributed to the enhancement of *n*_*s*_, which leads to a reduction in scattering cross-section as a result of screening. Increase in average kinetic energy of the electrons at the Fermi level leads to the reduction in scattering probability. However, the width of the region of electron confinement *b*_*f*_ also increases with *x* [see the inset of Fig. 3(b)]. This can result in an enhancement of the effective number of neutral impurity scattering centers. Therefore, a reduction in *μ*_*NI*_ is expected as *x* further increases. These two mutually opposite dependences on *x* can explain why *μ*_*NI*_(*x*) (and hence the overall mobility *μ*) goes through a peak. The origin for the decrease of *μ*_*PO*_ with the increase of *x* can be attributed to the involvement of greater number of subbands. More the number of subbands involved, higher is the rate of inter-subband scattering, which results in a reduction in *μ*_*PO*_ as the wall width gradually increases. Mobility as a function of temperature calculated at *x* = 0 and 5 are shown in Fig. 4(b,c), respectively. Contributions associated with individual scattering mechanisms are also shown as a function of temperature in these figures. Noticeably, the overall mobility, has a very weak temperature dependence. It is interesting to note that at *x* = 0 (tip of the wall) [see Fig. 4(b)], ionized impurity scattering is the most important mechanism governing the mobility whereas, at *x* = 5 [see Fig. 4(c)], the decisive contribution in determining the overall mobility comes from neutral impurity scattering at all temperatures (up to 300 K).

### GaN nanowall network: An example of polarization induced 2D confinement of carriers!

Recently, the growth of c-axis oriented wedge-shaped GaN nanowall network on sapphire substrates using molecular beam epitaxy technique has been reported^27^,^28^,^29^,^30^. These walls are found to be 100–200 nm wide at the base but less than 20 nm wide at the tip (see supplementary S-3). Interestingly, electron mobility in this material is estimated to be several orders of magnitude larger than that is observed in GaN bulk^28^,^31^,^32^. The material is found to be unintentionally n-type with electron concentration^31^,^32^ ≈10^19^ cm^−3^. Moreover, weak localization effect with a coherence length as long as 60 *μ*m, which is much larger than those reported for GaN/AlGaN heterostructure based two dimensional electron gas (2DEG) systems, has been observed in low temperature magnetotransport measurements^32^. Our study on these network structures furthermore reveals that the high electron mobility region extends down to several hundreds of nanometer below the tip of the walls^32^,^33^. This observation along with the finding of high mobility and long coherence length are suggestive of the fact that the electrons are 2D quantum confined in these walls. A brief report highlighting these experimental findings is provided as supplementary information S-3. Quantum confinement of carriers in this system is furthermore supported by the fact that photoluminescence and photoabsorption studies, which show as large as 150 meV blue shift of the band edge as the average tip-width of the walls decreases [see supplementary S-3]^34^. Note that our theory has estimated as high as 340 meV blue shift of the bandgap at the tip part of the wall as compared to bulk GaN [see Fig. 3(c)]. Since a wedge shaped wall has a varied thickness from top to bottom, one should expect a broad distribution of band gap associated with such a wall. In fact, both photoluminescence and photoabsorption studies carried out on GaN nanowall network samples show broad band-edge profiles as compared to those obtained for a epitaxial continuous films [see supplementary S-3]^31^,^33^,^34^. Electron mobility in these samples is found to decrease with the increase of the average tip width of the walls [see supplementary S-3]^32^,^34^. This result is consistent with our theory as shown in Fig. 4(a). Furthermore, the conductance shows hardly any temperature dependent variation in these samples [supplementary S-3]^32^, which is also in accordance with the results of Fig. 4(b,c). These findings thus strongly suggest that GaN nanowall network might be an example of polarization induced 2DEG formation.

Our study demonstrates a new way to achieve 2D carrier gas (2DCG) at the central plane of a wedged shaped wall structure made of a polar semiconductor. The advantage of the 2DCG formed this way over the conventional heterostructure based 2D systems is the significant suppression of ionized impurity scattering rate as a result of the natural separation of the carrier gas from the ionized impurities. An unique feature of this 2D channel is its ability to be formed in a plane perpendicular to the growth plane. One can envisage to fabricate large number of parallel 2D channels out of a single semiconducting film grown along its polarization direction using a suitable lithographic and etching technique. This may offer an opportunity for large scale integration of 2D devices in future.

## Additional Information

**How to cite this article**: Deb, S. *et al*. Polarization induced two dimensional confinement of carriers in wedge shaped polar semiconductors. *Sci. Rep.* **6**, 26429; doi: 10.1038/srep26429 (2016).

## Supplementary Material

Supplementary Information
