# Supplementary material for: Polarization induced two dimensional confinement of carriers in wedge shaped polar semiconductors
Source: Sci Rep. 2016 May 23;6:26429. doi: 10.1038/srep26429 (PMC4876402; doi:10.1038/srep26429)
Supplement: Supplementary Information [file srep26429-s1.pdf]

Polarization induced two dimensional confinement of carriers in wedge shaped polar semiconductors

S. Deb[1], H. P. Bhasker[1], Varun Thakur[2], S. M. Shivaprasad[2], and S. Dhar[1]

[1]*Department of Physics, Indian Institute of Technology Bombay, Powai, Mumbai 400076, India and*

[2]*International Centre for Material Science, Jawaharlal Nehru Centre for Advanced Scientific Research, Bangalore 560064, India*

S-1

Self consistent solution of Poisson's and Schrödinger equations

In order to solve the Poisson's and Schrödinger equations self consistently following iterative scheme has been adopted.

(i) A guess potential satisfying the boundary conditions is chosen. Schrödinger equation is solved for this potential to obtain the energy eigen values and eigenfunctions.

(ii) $n(x,z)$ and $N_d^+(x,z)$ are obtained.

(iii) Poisson's equation is solved using Gauss-Seidel iteration technique following C. F. Gerald[1] in a region, confined within a inclined facet [see Fig. 1]and the central plane ($z=0$) of the wall with the following boundary conditions:

1. At the top surface(x=0) : $\frac{\partial E_c(x,z)}{\partial x} = q_e \rho_s / 2\varepsilon_s \varepsilon_o \hat{n}$ where, $\rho_s = |\vec{P}|$ and $\hat{n} = \hat{x}$.
2. At the inclined facet : $\frac{\partial E_c(x,z)}{\partial x} = q_e \rho_s \cos\Theta / 2\varepsilon_s \varepsilon_o \hat{n}$ where, $\rho_s = |\vec{P}|\cos\Theta$ and $\hat{n} = \hat{x}$ and $\frac{\partial E_c(x,z)}{\partial z} = q_e \rho_s \sin\Theta / 2\varepsilon_s \varepsilon_o \hat{n}$ where, $\rho_s = |\vec{P}|\cos\Theta$ and $\hat{n} = \hat{z}$.
3. At the bottom surface (x=45 nm) : $\frac{\partial E_c(x,z)}{\partial x} = 0$.
4. At the central plane of the wall (z=0) : $E_c(x,0) = \mathrm{v_m}$.

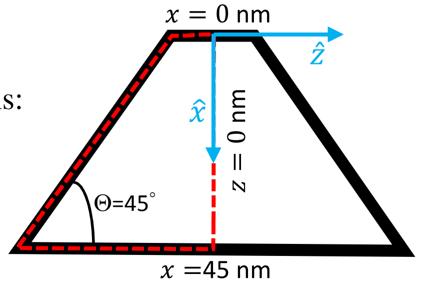

FIG. 1. Schematic view of the cross-Section of a wedge shape nanowall

At every step, profile for the other half of the trapezium has been obtained through $E_c(x,z) = E_c(x,-z)$ utilizing the symmetry property of the potential with respect to the $z=0$ plane.

(iv) Compare the new potential (conduction band profile) with that is obtained in the previous iteration. If both the profiles match within a specified tolerance limit, self consistency is achieved otherwise continue from step (i) with a revised guess potential formed with a certain linear combination of the newly obtained potential profile and the profile acquired at the previous iteration stage as given in Ref.

(v) Once self consistency is achieved, check the ratio (r_{ch}) between the total positive and negative charges developed within the wall (exclude the polarization charges). If r_{ch} is very close to 1 (within certain tolerance limit) stop the iteration. If not, then start the exercise all over again from step (i) with a revised value of $\mathrm{v_m}$. Note that $\mathrm{v_m}$ should be increased if $r_{ch} < 1$ and vice versa. The parameters used in this calculation are listed in Tab. 1.

TABLE I. Parameters used in this calculation.

Dielectric constant[2]	ε_s	8.9
	ε_∞	5.35
Electron effective mass[2]	m^*	0.2 m_e
Donor concentration	N_d	1×10^{19} cm^{-3}
Donor activation energy[3]	Δ	15 meV
Optical phonon energy[4]	$\hbar\omega$	0.092 eV
Band gap (300 K)[2]	E_g^{Bulk}	3.4 eV
Band gap (4 K)[5]	E_g^{Bulk}	3.5 eV
Spontaneous Polarization[6]	P	0.029 Cm^{-2}

[1] C. F. Gerald, *Applied Numerical Analysis*, Addison Wesley Publishing Company (1986)

[2] M. Shur, B. Gelmont and M. Asif Khan, J. of Elec. Mat **25**, 777 (1996)

[3] T. L. Tansley and R. J. Egan, Phys. Rev. B **45**, 10942 (1992)

[4] S. B. Lisesivdin, A. Yildiz, N. Balkan, M. Kasap, S. Ozcelik and E. Ozbay, J. Appl. Phys. **108**, 013712 (2010)

[5] H. Morkoc, *Nitride Semiconductors and Devices*, Springer (1998)

[6] E. T. Yu, X. Z. Dang, P. M. Asbeck, S. S. Lau and G. J. Sullivan, J. Vac. Sci. Technol. B **17**, 1742 (1999)

S-2

Calculation of mobility limited by different scatterings

Momentum relaxation time for ionized impurity (intrasubband) scattering of the electrons belonging to the m-th subband of a 2DEG can be expressed as[1,2]

$$\frac{1}{\tau_x^{II}(E_m)} = \frac{m^* e^4}{8\pi\hbar^3\varepsilon_s^2\varepsilon_o^2}\int_0^{2\pi}\int_0^{\infty}\frac{1}{q^2\varepsilon_{mm}^2(q)}v_x^2(q,z_i)(1-\cos\theta)N_d^+(x,z_i)d\theta dz_i \tag{1}$$

where, $v_x(q,z_i)$= $\int_{-b/2}^{+b/2}|\zeta_x^{(m)}(z)|^2 e^{-q|z-z_i|}dz$, $\vec{q} = \vec{k'} - \vec{k}$ with $\vec{k}$ and $\vec{k'}$ be the initial and final k-state of an electron after scattering, θ the angle between $\vec{k}$ and $\vec{k'}$ and z_i the z-coordinate position of the scatterer. It should be noted that the relaxation time $\tau_x^{II}(E_m)$ has a week x dependence as a result of the slow variation of the wave function $\zeta_x^{(m)}(z)$ with x. The screening factor $\varepsilon_x^{mm}(q)$ = $1+\left[e^2m^*/2\pi\hbar^2\varepsilon_o\varepsilon_s q\right]\sum_i F_x^{ii,mm}(q)f(\mathbb{E}_i)$,[2–4] where the form factor can be expressed as $F_x^{ij,mn}(q)$ = $\int_{-b/2}^{+b/2}\int_{-b/2}^{+b/2}\zeta_x^{(n)*}(z)\zeta_x^{(m)}(z)\zeta_x^{(i)*}(z')\zeta_x^{(j)}((z'))e^{-q|z-z'|}dzdz'$, i,j,m and n stand for different subbands, $q = 2k\sin(\theta/2)$ as the ionized impurity scattering is an elastic process.

In order to calculate the neutral impurity scattering rate, we have adopted the general scheme for calculating the rate of any elastic scattering event in 2D system proposed by Stren *et al.*[5]. In general, momentum cross-section due to any spherically symmetric potential $U(r)$ for the electrons belonging to the m-th subband of a 2D system can be written as:[5]

$$\sigma^{NI}(E_m) = \frac{2m^{*2}\pi}{k\hbar^4}\left|\int_0^{\infty}U(r)J_0(qr)rdr\right|^2\int_0^{2\pi}(1-\cos\theta)d\theta \tag{2}$$

where, $\vec{r}$ represents the position vector in 2D, $J_0(qr) = \sqrt{2/\pi qr}\cos(qr-\pi/4)$. Here we have treated the neutral impurity potential as a spherically symmetric square well[6]; $U(r)$=Δ when $r \leq a_o$ and $U(r)$=0 when $r > a_o$. Where, a_o the effective Bohr radius of an electron on a shallow donor. Finally, the momentum relaxation time can be obtained as

$$\frac{1}{\tau_x^{NI}(E_m)} = \frac{\hbar k\sigma^{NI}(E_m)\int_{-b/2}^{+b/2}N_u(x,z)dz}{m^*} \tag{3}$$

where, $N_u(x,z)$ = $[N_d - N_d^+(x,z)]n(x,z)/n(x,0)$ is the weighted concentration of neutral donors. At high temperatures, the major contribution to the scattering arises due to the lattice vibration. However it is noteworthy that the rate of scattering due to polar phonon (PO) is expected to be much higher than that for the deformation and piezoelectric potential scattering in GaN because

of its strong polar nature.[7] Following Ridley[8], we write the momentum relaxation time limited by PO phonon scattering as:

$$\frac{1}{\tau_x^{PO}(E_m)} = \mathbb{G}_{\mp}(k)\sum_{n=1}^{n_m}\left(2+\delta_{mn} - \frac{(1+\delta_{mn})\left(\left(\sqrt{\mathbb{E}_m}-\sqrt{\mathbb{E}_n}\right)^2 \pm \mathbb{Z}_{\pm}\right)}{\sqrt{d_{1\pm}}} - \frac{\left(\sqrt{\mathbb{E}_m}+\sqrt{\mathbb{E}_n}\right)^2 \pm \mathbb{Z}_{\pm}}{\sqrt{d_{2\pm}}}\right) \tag{4}$$

where,

$$\mathbb{G}_{\mp}(k) = e^2\omega\left(N(\omega)+\frac{1}{2}\mp\frac{1}{2}\right)/(32\pi b_f(x)\mathcal{E}_k\varepsilon_o\varepsilon_p)$$

$$\mathbb{Z}_{\pm} = \hbar\omega \pm (\mathbb{E}_m - \mathbb{E}_n)$$

$$\varepsilon_p^{-}1 = \varepsilon_\infty^{-1} - \varepsilon_s^{-1}$$

$$d_{1\pm} = 2\left(\sqrt{\mathbb{E}_m}-\sqrt{\mathbb{E}_n}\right)^2(\mathcal{E}_k \pm \mathbb{Z}_{\pm}) + \mathbb{Z}_{\pm}^2 + \left(\sqrt{\mathbb{E}_m}-\sqrt{\mathbb{E}_n}\right)^4$$

$$d_{2\pm} = 2\left(\sqrt{\mathbb{E}_m}+\sqrt{\mathbb{E}_n}\right)^2(\mathcal{E}_k \pm \mathbb{Z}_{\pm}) + \mathbb{Z}_{\pm}^2 + \left(\sqrt{\mathbb{E}_m}+\sqrt{\mathbb{E}_m}\right)^4$$

$$N(\omega) = \left(e^{\hbar\omega/k_BT}-1\right)^{-1}$$

where, the upper and lower signs have been used for absorption and emission respectively. Value of n_m, can be evaluated from energy conservation $\mathbb{E}_{n_m} = E_m \pm \hbar\omega$. From eqn. 4 it may appear that the contribution of emission processes is more than that of absorption processes as the factor $(N(\omega)+1)$ associated with the emission is clearly larger than the factor $N(\omega)$ for absorption. However, it should be noted that PO phonon energy in GaN is $\approx$ 90 meV, which is much larger than k_BT even at room temperature. In a degenerate 2D electron gas, the possibility of transition accompanied by phonon emission is expected to be feeble because of unavailability of states below $(E_F - k_BT)$. Here we have thus ignored the scattering processes involving emission of PO phonon.

---

1 D. Chattopadhyay and H. J. Queisser, Rev. of Mod. Phys. **53**, 745 (1981)

2 M. Dür, S. M. Goodnick and P. Lugli, Phys. Rev. B **54**, 17794 (1996)

3 D. K. Ferry and S. M. Goodnick, *Transport in Nanostructures*, Cambridge University Press (2001)

4 T. Ando, J. Phys. Soc. Jpn. **51**, 3900 (1982)

5 F. Stren and W. E. Howard, Phys. Rev. **163**, 816 (1967)

6 B. K. Ridley, *Quantum Processes in Semiconductors*, Oxford University Press (1999)

7 M. Shur, B. Gelmont and M. Asif Khan, J. of Elec. Mat **25**, 777 (1996)

[8] B. K. Ridley, J. Phys. C: Solid State Phys. **15**, 5899 (1982)

S-3

GaN nanowall network: An example of polarization induced 2D confinement of carriers

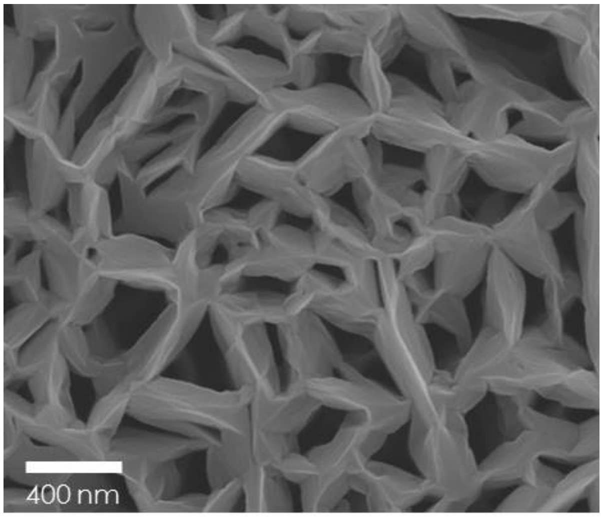

FIG. 2. Top view SEM of a GaN nanowall network sample. Wedge shaped wall structures forming a network is noticeable.[1–6]

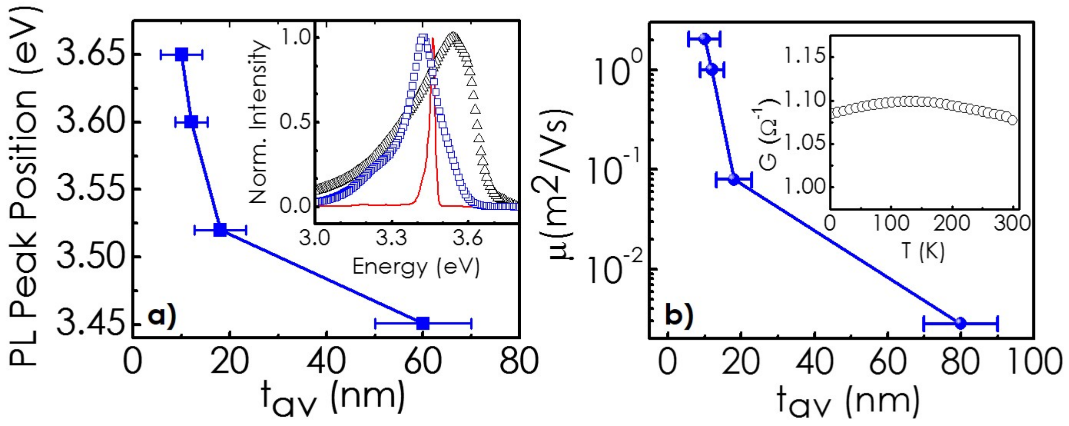

FIG. 3. (a) Position of PL peak as a function of the average tip width of the network (t_{av}). Inset shows the near band edge PL spectra recorded at 10 K for two different samples with the average value of the tip width t_{av} to be 12±3 nm (Sample B; black open triangle) and 18±5 nm (Sample C; blue open rectangle), respectively. 10 K PL spectrum (red solid line) for a reference GaN epitaxial continuous film is also shown for comparison. Clearly, the spectrum for sample B is blue shifted as compared to that for the sample C and the reference sample. Moreover, the profiles for the nanowall network samples are much broader (both at high and low energy regions) than that for the reference sample [see Ref. 6 for more detail]. (b) Experimentally estimated mobility as a function of t_{av} [see Ref. 4 and 5 for more detail]. Inset shows the temperature variation of conductance G measured from 1.5 to 300 K for sample B. Note that the G hardly varies (change is $< 1\%$) as the temperature increases from 1.5 to 300 K.

---

[1] M. Kesaria, S. Shetty and S. M. Shivaprasad, Cryst. Growth. Des. **11**, 4900 (2011)

[2] M. Kesaria and S. M. Shivaprasad, Appl. Phys. Lett. **99**, 143105 (2011)

[3] A. Zhong and K. Hane, Japan. J. Appl. Phys. **52**, 08JE13 (2013)

[4] H. P. Bhasker, S. Dhar, A. Sain, Manoj Kesaria and S. M. Shivaprasad, Appl. Phys. Lett. **101**, 132109 (2012)

[5] H. P. Bhasker, Varun Thakur, S. M. Shivaprasad and S. Dhar, J. Phys. D: Appl. Phys. **48**, 255302 (2015)

[6] H. P. Bhasker, Varun Thakur, Manoj Kesaria,S. M. Shivaprasad and S. Dhar, AIP Conf. Proc. **1583**, 252 (2014)
